# Promotional Tone in Reviews of Menopausal Hormone Therapy After the Women's Health Initiative: An Analysis of Published Articles

**DOI:** 10.1371/journal.pmed.1000425

**Published:** 2011-03-15

**Authors:** Adriane Fugh-Berman, Christina Pike McDonald, Alicia M. Bell, Emily Catherine Bethards, Anthony R. Scialli

**Affiliations:** 1 Department of Physiology and Biophysics, Georgetown University Medical Center, Washington D.C., United States of America; 2 Department of Obstetrics and Gynecology, Georgetown University Medical Center, Washington D.C., United States of America; York University, Canada

## Abstract

**Background:**

Even after the Women's Health Initiative (WHI) found that the risks of menopausal hormone therapy (hormone therapy) outweighed benefit for asymptomatic women, about half of gynecologists in the United States continued to believe that hormones benefited women's health. The pharmaceutical industry has supported publication of articles in medical journals for marketing purposes. It is unknown whether author relationships with industry affect promotional tone in articles on hormone therapy. The goal of this study was to determine whether promotional tone could be identified in narrative review articles regarding menopausal hormone therapy and whether articles identified as promotional were more likely to have been authored by those with conflicts of interest with manufacturers of menopausal hormone therapy.

**Methods and Findings:**

We analyzed tone in opinion pieces on hormone therapy published in the four years after the estrogen-progestin arm of the WHI was stopped. First, we identified the ten authors with four or more MEDLINE-indexed reviews, editorials, comments, or letters on hormone replacement therapy or menopausal hormone therapy published between July 2002 and June 2006. Next, we conducted an additional search using the names of these authors to identify other relevant articles. Finally, after author names and affiliations were removed, 50 articles were evaluated by three readers for scientific accuracy and for tone. Scientific accuracy was assessed based on whether or not the findings of the WHI were accurately reported using two criteria: (1) Acknowledgment or lack of denial of the risk of breast cancer diagnosis associated with hormone therapy, and (2) acknowledgment that hormone therapy did not benefit cardiovascular disease endpoints. Determination of promotional tone was based on the assessment by each reader of whether the article appeared to promote hormone therapy. Analysis of inter-rater consistency found moderate agreement for scientific accuracy (κ = 0.57) and substantial agreement for promotional tone (κ = 0.65). After discussion, readers found 86% of the articles to be scientifically accurate and 64% to be promotional in tone. Themes that were common in articles considered promotional included attacks on the methodology of the WHI, arguments that clinical trial results should not guide treatment for individuals, and arguments that observational studies are as good as or better than randomized clinical trials for guiding clinical decisions. The promotional articles we identified also implied that the risks associated with hormone therapy have been exaggerated and that the benefits of hormone therapy have been or will be proven. Of the ten authors studied, eight were found to have declared payment for speaking or consulting on behalf of menopausal hormone manufacturers or for research support (seven of these eight were speakers or consultants). Thirty of 32 articles (90%) evaluated as promoting hormone therapy were authored by those with potential financial conflicts of interest, compared to 11 of 18 articles (61%) by those without such conflicts (*p* = 0.0025). Articles promoting the use of menopausal hormone therapy were 2.41 times (95% confidence interval 1.49–4.93) as likely to have been authored by authors with conflicts of interest as by authors without conflicts of interest. In articles from three authors with conflicts of interest some of the same text was repeated word-for-word in different articles.

**Conclusion:**

There may be a connection between receiving industry funding for speaking, consulting, or research and the publication of promotional opinion pieces on menopausal hormone therapy.

*Please see later in the article for the Editors' Summary*

## Abstract 2

Adriane Fugh-Berman and colleagues analyzed a selection of published opinion pieces on hormone therapy and show that there may be a connection between receiving industry funding for speaking, consulting, or research and the tone of such opinion pieces.

## Editors' Summary

**Background:**

Over the past three decades, menopausal hormones have been heavily promoted for preventing disease in women. However, the Women's Health Initiative (WHI) study—which enrolled more than 26,000 women in the US and which was published in 2004—found that estrogen-progestin and estrogen-only formulations (often prescribed to women around the age of menopause) increased the risk of stroke, deep vein thrombosis, dementia, and incontinence. Furthermore, this study found that the estrogen-progestin therapy increased rates of breast cancer. In fact, the estrogen-progestin arm of the WHI study was stopped in 2002 due to harmful findings, and the estrogen-only arm was stopped in 2004, also because of harmful findings. In addition, the study also found that neither therapy reduced cardiovascular risk or markedly benefited health-related quality of life measures.

Despite these results, two years after the results of WHI study were published, a survey of over 700 practicing gynecologists—the specialists who prescribe the majority of menopausal hormone therapies—in the US found that almost half did not find the findings of the WHI study convincing and that 48% disagreed with the decision to stop the trial early. Furthermore, follow-up surveys found similar results.

**Why Was This Study Done?:**

It is unclear why gynecologists and other physicians continue to prescribe menopausal hormone therapies despite the results of the WHI. Some academics argue that published industry-funded reviews and commentaries may be designed to convey specific, but subtle, marketing messages and several academic analyses have used internal industry documents disclosed in litigation cases. So this study was conducted to investigate whether hormone therapy–promoting tone could be identified in narrative review articles and if so, whether these articles were more likely to have been authored by people who had accepted funding from hormone manufacturers.

**What Did the Researchers Do and Find?:**

The researchers conducted a comprehensive literature search that identified 340 relevant articles published between July 2002 and June 2006—the four years following the cessation of the estrogen-progestin arm of the women's health initiative study. Ten authors had published four to six articles, 47 authored two or three articles, and 371 authored one article each. The researchers focused on authors who had published four or more articles in the four-year period under study and, after author names and affiliations were removed, 50 articles were evaluated by three readers for scientific accuracy and for tone. After individually analyzing a batch of articles, the readers met to provide their initial assessments, to discuss them, and to reach consensus on tone and scientific accuracy. Then after the papers were evaluated, each author was identified and the researchers searched for authors' potential financial conflicts of interest, defined as publicly disclosed information that the authors had received payment for research, speaking, or consulting on behalf of a manufacturer of menopausal hormone therapy.

Common themes in the 50 articles included arguments that clinical trial results should not guide treatment for individuals and suggestions that the risks associated with hormone therapy have been exaggerated and that the benefits of hormone therapy have been or will be proven. Furthermore, of the ten authors studied, eight were found to have received payment for research, speaking or consulting on behalf of menopause hormone manufacturers, and 30 of 32 articles evaluated as promoting hormone therapy were authored by those with potential financial conflicts of interest. Articles promoting the use of menopausal hormone therapy were more than twice as likely to have been written by authors with conflicts of interest as by authors without conflicts of interest. Furthermore, Three authors who were identified as having financial conflicts of interest were authors on articles where sections of their previously published articles were repeated word-for-word without citation.

**What Do These Findings Mean?:**

The findings of this study suggest that there may be a link between receiving industry funding for speaking, consulting, or research and the publication of apparently promotional opinion pieces on menopausal hormone therapy. Furthermore, such publications may encourage physicians to continue prescribing these therapies to women of menopausal age. Therefore, physicians and other health care providers should interpret the content of review articles with caution. In addition, medical journals should follow the International Committee of Medical Journal Editors Uniform Requirements for Manuscripts, which require that all authors submit signed statements of their participation in authorship and full disclosure of any conflicts of interest.

**Additional Information:**

Please access these Web sites via the online version of this summary at http://dx.doi.org/10.1371/journal.pmed.1000425.

- The US National Heart, Lung, and Blood Institute has more information on the Womens Health Initiative
- The US National Institutes of Health provide more information about the effects of menopausal hormone replacement therapy
- The Office of Women's Health, U.S. Department of Health and Human Services provides information on menopausal hormone therapy
- The International Committee of Medical Journal Editors Uniform Requirements for Manuscripts presents Uniform Requirements for Manuscripts published in biomedical journals
- The National Womens Health Network, a consumer advocacy group that takes no industry money, has factsheets and articles about menopausal hormone therapy
- PharmedOut, a Georgetown University Medical Center project, has many resources on pharmaceutical marketing practices

## Introduction

About half of US gynecologists continue to distrust the results of the Women's Health Initiative (WHI) [1],[2],[3], which found that risks of menopausal hormone therapy outweighed benefits in asymptomatic women. Such resistance to the findings of the largest randomized, placebo-controlled trial of menopausal hormone therapy ever performed is curious.

The WHI enrolled more than 26,000 women. After more than five years of follow-up, the estrogen-progestin arm of the WHI was stopped in 2002 due to harm; the estrogen-only arm was stopped in 2004, also due to harm [4],[5]. Both therapies increased the risk of stroke, deep vein thrombosis [4],[5], dementia [6], and incontinence [7]; estrogen-progestin therapy also increased rates of breast cancer [4]. Neither therapy reduced cardiovascular risk, and neither markedly benefited health-related quality of life measures [8],[9].

Over the year after the first WHI results were announced, hormone prescriptions dropped by 80% [10]. Compared to 2002, the age-adjusted incidence of breast cancer diagnosis dropped 6.7% in 2003, a finding attributed to decreased use of hormone therapy among postmenopausal women [11],[12]. A recent report confirmed an increased breast cancer incidence and a doubling of breast cancer-associated deaths among hormone users in the WHI [13].

In the US, gynecologists were more likely than internists or family physicians to prescribe hormone therapy to asymptomatic women both before [14],[15] and after [16],[17] the WHI. Between 1999 and 2002, 90 million hormone therapy prescriptions were written annually [18]. In 2002, US gynecologists wrote 70% of estrogen or estrogen-progestin prescriptions [19]. Sixteen months after the WHI ended, a survey of 705 fellows of the American College of Obstetricians and Gynecologists found that 49.1% of gynecologists did not find the WHI convincing and that 48.1% disagreed with the decision to stop the trial [1]. Follow-up surveys found similar results [2],[3].

Publications in the medical literature by industry-paid physicians have recently received attention [20]. Several academic analyses have used internal industry documents disclosed in litigation to document the use of messaging in publications regarding rofecoxib (Vioxx) [21], gabapentin (Neurontin) [22], and sertraline (Zoloft) [23]. Thousands of internal documents are available at the Drug Industry Document Archive (http://dida.library.ucsf.edu/). Industry-funded reviews and commentaries may be designed to convey specific, but subtle, marketing messages [24]. As a possible explanation for why so many physicians continue to support the use of menopausal hormone therapy in asymptomatic women, we investigated whether promotional tone could be identified in narrative review articles regarding menopausal hormone therapy and whether articles identified as promotional were more likely to have been authored by those with conflicts of interest as determined by declared payments from hormone manufacturers.

## Methods

### Search Strategy

In 2006, we conducted a MEDLINE search using Ovid, limited to English language reviews or comments published from July 2002 through June 2006 (the four years after cessation of the estrogen-progestin arm of the WHI). The search terms were “estrogen replacement therapy” combined with “breast neoplasms AND menopause” or “cardiovascular disease AND menopause”; “hormone replacement therapy” combined with “breast neoplasms AND menopause” or “cardiovascular disease AND menopause”; and “menopausal hormone therapy” combined with “breast cancer.” A list of all authors was made and the number of publications per author assessed. A subset of the most prolific authors was identified. One reader (CPM) assessed article titles, eliminating articles that did not focus on benefits and risks of combined estrogen-progestin menopausal hormone therapy since the WHI. An additional MEDLINE search was conducted using the names of these authors to identify other relevant articles.

### Analysis of Content

Articles were obtained, and information on identifying authors and affiliations was removed, by support staff. All articles were independently evaluated for scientific accuracy and for tone by three readers (CPM, ECB, AMB) who were then graduate students in physiology. We used graduate students in physiology as readers because these individuals have the requisite knowledge to understand the technical aspects of the literature but have no experience as health care providers or as the targets of pharmaceutical promotional efforts. Scientific accuracy was assessed based on whether or not the findings of the WHI were accurately reported using two criteria:

1. Acknowledgment or lack of denial of the risk of breast cancer diagnosis associated with menopausal hormone therapy, and
2. Acknowledgment that menopausal hormone therapy did not benefit cardiovascular disease endpoints.

Determination of tone was based on each reader's assessment of whether the article appeared to promote hormone therapy (such articles were deemed “promotional”) or not (articles deemed “nonpromotional” were either neutral about hormone therapy or argued against routine use). Because no validated tools exist for assessing tone, each reader made her determination according to personal criteria. Readers were asked to record features of each article supporting their assessments. Assessments were discussed only at team meetings, and readers did not otherwise communicate with each other about the articles.

The initial literature search identified 340 articles with a total of 428 authors. Ten authors had published four, five, or six articles; 47 authored two or three articles; and 371 authored one article each. We then excluded articles that focused on selective estrogen receptor modulators, androgens, specific receptors, or specific mechanisms, and other articles unlikely to discuss the risks and benefits of menopausal hormone therapy. After excluding these articles, 232 potentially relevant articles remained.

The ten authors who had published four to six articles accounted for 47 (20%) of these articles. We chose to focus on these ten authors because they contributed a fifth of the extant literature during the four-year period under study, and could be assumed to have been widely read and thus influential. An additional literature search using these author names identified 13 additional relevant articles for a total of 60 articles for blinded evaluation by the three readers. Ten articles were subsequently excluded by mutual agreement of all readers (two publications were letters, three were duplicated references, four covered topics outside the scope of the study and did not specifically address our criteria, and one was published before July 2002). The remaining 50 papers, listed in Text S1, were evaluated individually by all readers. The ten authors who published four or more articles in the four-year period under study are identified in Table 1.

**Table 1:** Scientific accuracy and promotional tone in articles on menopausal hormone therapy.

		Articles authored or coauthored by authors with potential financial conflicts of interest^a^ (41 total)	Articles authored or coauthored by authors without potential financial conflicts of interest^b^ (9 total)
Scientific accuracy	No	6^c^	1^d^
	Yes	35^e^	8^f^
Promotional tone	No	11^g^	7^h^
	Yes	30^i^	2^j^

a Authors with potential financial conflicts of interest: Leon Speroff, Thomas B. Clarkson, Peter Kenemans, Rogerio A. Lobo, David M. Herrington, Marius J. van der Mooren, Rowan T. Chlebowski, Susan R. Davis.

b Authors with no evidence of potential financial conflicts of interest: Nanette K. Wenger, Nananda F. Col.

The following footnotes indicate references listed in Text S1.

c A10, A12, A25, A29, A35, A45.

d A4.

e A1-2, A5, A8-9, A11, A14, A15-24, A26-27, A30-34, A36-38, A40-43, A46-48, A50.

f A3, A6-7, A13, A28, A39, A44, A49.

g A1-2, A16-17, A22, A24-25, A32, A46, A47-48.

h A6-7, A13, A28, A39, A44, A49.

i A5, A8, A9-12, A14-15, A18-21, A23, A26-27, A29-31, A33-38, A40-43, A45, A50.

j A3, A4.

After individually analyzing a batch of articles, readers met to provide their initial assessments, to discuss them, and to come to consensus, if possible, on scientific accuracy and tone. Disagreements that could not be resolved in discussion were resolved by majority vote. After all articles had been evaluated individually and together by all readers, the authors were revealed and a search was performed for potential financial conflicts of interest, defined as evidence that the authors had received payment for research, speaking, or consulting on behalf of a manufacturer of menopausal hormone therapy.

### Assessment of Conflicts of Interest

Conflicts of interest were assessed by examining published declarations within MEDLINE-indexed articles, disclosures from the Council on Hormone Education (a Wyeth-funded group of consultants) [25]–[28], and a Google search of each author's name combined with the term “conflict of interest.” Conflicts of interest were assessed for the time period ending 30 June 2006. No evaluations of potential conflicts of interest disclosed after that date were attempted.

### Statistical Analysis

The Fisher exact test was used to assess the distribution of articles between conflicted and nonconflicted authors. A two-sided *p*-value of 0.05 or less was accepted as statistically significant. Risk ratios were estimated using the SABER program from the US Centers for Disease Control and Prevention. The kappa statistic (κ) was calculated according to the method of Brennan and Prediger [29].

## Results

The majority of articles (86%) were judged to be scientifically accurate according to our analysis. Thirty-two (64%) of the 50 articles were assessed as promotional.

After individual evaluation, all three readers agreed on both scientific accuracy and tone for half (50%) of the articles. Readers were in agreement regarding scientific accuracy for 34 articles (68%) and regarding promotional tone for 39 articles (78%). Analysis of inter-rater consistency found moderate agreement for scientific accuracy (κ = 0.57) and substantial agreement for promotional tone (κ = 0.65). After the consensus discussions, there was agreement regarding scientific accuracy for 49 articles (98%) and regarding promotional tone for 48 (96%) articles. Overall, readers were in 94% agreement prior to unmasking the identity of the authors.

### Themes in Promotional Articles

Readers identified themes common among promotional articles. These themes included:

- The WHI was flawed.
- The WHI was a controversial trial.
- The population studied in the WHI was inappropriate or was not representative of the general population of menopausal women.
- Clinical trial results should not guide treatment for individuals.
- Observational studies are as good as or better than randomized clinical trials.
- Animal studies can guide clinical decision-making.
- The risks associated with hormone therapy have been exaggerated.
- The benefits of hormone therapy have been or will be proven; recent studies are an aberration.

For examples of statements representative of the themes identified, see Table 2. Examples of what were considered promotional and nonpromotional treatments of similar topics are given in Table 3.

**Table 2:** Themes consistently identified in promotional articles, with examples.

Theme	Examples^a^
The Women's Health Initiative was flawed.	“Will postmenopausal hormone therapy begun at or near the time of the menopause, and maintained for a relatively long duration of time, provide protection against coronary artery disease (primary prevention)? The design of the canceled arm of the WHI did not allow an answer to this question.” (A10, A50)
The Women's Health Initiative was a controversial trial.	“The proper role of hormone replacement therapy (HRT) for the treatment and prevention of cardiovascular disease remains a controversial and heavily debated topic.” (A32)“… we cannot formulate any general advice that holds for the majority of European post-menopausal women due to lack of consistency, lack of biological plausibility, and lack of relevance of randomized clinical trial data to our daily practical work.” (A11)“Therefore, the WHI results are questionable and uncertain even if obtained in a gold standard randomized clinical trial, and for sure these data cannot be extrapolated to the younger and healthier perimenopausal women.” (A35)
The population studied in the Women's Health Initiative was inappropriate or was not representative of the general population of menopausal women.	“In the three large RCTs available, the populations studied are: not representative, too old and without climacteric complaints, and therefore lacking any indication for postmenopausal hormone therapy (HT).” (A11)“Randomized controlled trials are very powerful investigative tools that are, by design, limited in their interpretation to populations covered by the randomized controlled trial.” (A43)
Clinical trial results should not guide treatment for individuals.	“To do as the editorialists recommend (to categorically discourage the use of hormone therapy) is to deny women the assistance they need to make individual decisions based upon individual characteristics and needs.” (A8)
Observational studies are as good or better than randomized clinical trials.	“However, a truly randomized, placebo-controlled, HT trial in women entering menopause is very difficult or even impossible to carry out in practice and observational studies are more akin to the approach of physicians in clinical practice.” (A31)
Animal studies can guide clinical decision-making.	“There is strong evidence that ET/HT provides cardioprotection in primary prevention studies of animal models with supportive data from studies of women in the EPAT and Nurses' Health Study.” (A27)
The risks associated with hormone therapy have been exaggerated.	“The important unanswered question is whether hormone therapy causes breast cancer or is promoting the diagnosis of pre-existing tumors.” (A8)“It is also worth pointing out that the reported risk with hormone therapy is even smaller than that associated with recognized risk factors such as a positive family history, being overweight after menopause, and alcohol intake.” (A10, A30, A50)“However, this proportion was not due to an epidemic of breast cancer in the HRT group but rather to an unexplained decreased incidence of breast cancer cases in the placebo group.” [35]“It is helpful to emphasize the possibility that the studies reflect an effect of hormone therapy on preexisting tumors[,] and that hormone users who develop breast cancer have a reduced risk of dying of breast cancer because their tumors are better differentiated, more localized, and smaller.” (A10, A30, A50)
The benefits of hormone therapy have been or will be proven; recent studies are an aberration.	“Mortality from all causes is lower in HRT users compared with non-users.” (A12)“However, even after the most contemporary and careful attempts to adjust for possible confounders, HRT use continues to be associated with more favorable outcomes.” (A15)“With many observational trials indicating a cardioprotective effect of early estrogen treatment and the absence of a prospective, randomized clinical trial powered to reveal cardioprotection starting during the menopausal transition it seems prudent not to dismiss such an effect.” (A43)“There continues to be good reason to believe that there are benefits associated with treatment, including improvement of quality of life beyond the relief of hot flushes, maximal protection against osteoporotic fractures, a reduction in colorectal cancers, maintenance of skin turgor and elasticity, and the possibility of primary prevention of CHD and Alzheimer's disease.” (A10)

a See Text S1 for referenced articles given in parenthesis.

**Table 3:** Examples of language promoting or not promoting the use of hormone therapy.

Promotional	Nonpromotional
“…the WHI did not study the appropriate population in the appropriate time period to establish that hormone therapy does not exert a primary preventive effect on the risk of CHD.” (A10, A50)	“The role of conventional HRT for treatment and prevention of CHD is rapidly evolving from presumed benefit to proven harm.” (A46)
“Are such experts jumping to conclusions, and jeopardizing the health and quality of life of these peri- and early postmenopausal women, who differ from those in the WHI trial, two-thirds of whom were over 60 years old?” (A18)	“…use of menopausal hormone therapy (MHT) for chronic disease risk reduction in any population cannot be supported.” (A47)
“Wherever we live we are surrounded by risks, sometimes not known to us. Air and water pollution, various forms of radiation, insecticides in food and road accidents are good examples. Do we stop using the car? Certainly not! We take the small risk because of the benefits.” (A35)	“Although the majority of WHI women had no adverse events, the population risk is substantial, such that the global risk: benefit profile does not warrant recommendation of this therapy as a widespread preventive intervention.” (A7)
“Given the practical challenges in conducting randomized controlled trials to compare the various estrogens, drawing inferences from valid observational studies seems the only realistic alternative.” (A3)	“Observational studies have inherent biases that cannot be corrected for; therefore evidence should come from randomized clinical trials (RCTs).” (A11)
“The use of animal models allows for the evaluation of direct breast cancer effects of ET [estrogen therapy] and HT and avoids some of the confounding variables that are necessarily a part of studies on postmenopausal women.” (A38)	“…observational or mechanistic studies, animal models, and basic research have tremendous value for the generation of hypotheses but should not be used to justify broad-based pharmacologic interventions.” (A5)

See Text S1 for referenced articles given in parenthesis.

Articles evaluated as promotional frequently differed from nonpromotional articles in the way that risks were presented. For example, most articles conceded that hormone therapy was associated with breast cancer, but promotional articles contained statements such as “The risk of breast cancer with hormonal therapy is put into perspective with the realization that this risk is related to hormonal dose and duration of use, and that the absolute risk remains small” (reference A14 in Text S1), or “The WHI agreed with convincing evidence in the literature that postmenopausal hormone therapy does not increase the risk of breast cancer beyond that already associated with recognized risk factors, such as a positive family history” (A10 in Text S1). A nonpromotional article stated “Although estrogen and/or progestin effectively reduce vasomotor symptoms, a recent WHI randomized trial identified an unfavorable risk/benefit balance on life-threatening diseases, including increased breast cancer, for combined estrogen plus progestin use in otherwise healthy post-menopausal women” (A40 in Text S1). See Table 3 for more examples of statements that minimize risks.

### Reuse of Previously Published Text

Our analysis also found, incidentally, that three authors we identified as having financial conflicts of interest were authors on articles where sections of their previously published articles were repeated word-for-word. Neither of the authors without declared conflicts of interest were noted to have reused text in the articles analyzed.

Eighty-four percent of the text and all seven tables in one article (A9 in Text S1) were found in four other articles by Leon Speroff (A8, A10, A30, and A50 in Text S1). Seventy-three percent of the text and seven of ten tables from one of these articles (A10 in Text S1) were found in three other articles by this author (A9, A30, and A50 in Text S1). More than half (55%) of the text of one article (A8 in Text S1), published in *Maturitas*, was reused in the same journal a year later (A9 in Text S1). No acknowledgment of earlier publication was made.

Twenty-five percent of an article coauthored by Susan R. Davis was reused (A25 in Text S1) in another article coauthored by the same author, without acknowledgment of earlier publication (A26 in Text S1). Both articles included additional authors and we could not determine which authors were responsible for the reuse. Over two-thirds (71%) of the text of an article by Rogerio A. Lobo (A33 in Text S1), as well as its two figures and table, were reused (A14 in Text S1); in this case, adaptation, but not republication, was acknowledged (A14 in Text S1).

### Conflicts of Interest

Of the ten authors in our sample, eight were found to have received payment for research, speaking, or consulting on behalf of menopause hormone manufacturers (Table 1). About half of the articles analyzed had coauthors in addition to the ten authors we assessed, but we did not examine whether there were potential financial conflicts of interest for these coauthors.

The assessments of scientific accuracy and promotional tone after the consensus discussion are summarized in Table 1 according to author's potential conflict of interest. Thirty of 32 articles (90%) evaluated as promoting hormone therapy were authored by those with conflicts of interest compared to 11 of 18 articles (61%) by those without conflicts of interest. The difference was significant (*p* = 0.0025). Two of nine articles (22%) by authors without known conflicts were considered promotional, and 30 of 41 articles (73%) by authors with known conflicts were considered promotional. Articles promoting the use of menopausal hormone therapy were 2.41 times (95% CI 1.49–4.93) more likely to have been written by authors with conflicts of interest than by authors without conflicts of interest.

## Discussion

This study evaluated the relationship between the receipt by authors of payment from industry for speaking or consulting and authorship of articles considered to be scientifically in error or promotional in tone. We identified an association of review articles promoting the use of hormone therapy with authors with declared financial conflicts of interest. Scientific accuracy did not appear to be affected by author conflicts of interest.

The effect of industry funding on results in clinical trials [30], meta-analyses [31],[32], clinical practice guidelines [33], and pay-for-performance quality measures [34] has been well documented. Two publications have documented scientifically unsupportable statements on the risks and benefits of menopausal hormone therapy in the medical literature [19],[35]; many of these statements appeared in reviews, commentaries, editorials, and letters.

We assessed scientific accuracy based on whether or not two findings of the WHI were accurately reported: (1) There is no proven cardioprotective effect of estrogen-progestin therapy in menopausal women or in ten-year age subgroups of menopausal women, and (2) breast cancer is diagnosed more frequently in menopausal women receiving estrogen-progestin therapy [4],[36]. Promotional tone was evaluated without formal criteria, but the readers were asked to identify elements of the articles that conveyed a promotional tone. Readers evaluated articles masked as to the identity or affiliations of the authors. Prior to discussion, there was substantial agreement among the individual readers for promotional tone, and moderate agreement on scientific accuracy. After discussion, but prior to unmasking of author identities, there was consensus on both measures for all but two of the 50 papers evaluated.

We found that articles with a promotional tone were more likely to have been written by authors who had disclosed financial conflicts of interest than by authors without such disclosures. These conflicts were determined through publicly available declarations of conflicts of interest and may not be accurate or complete. It is possible, for example, that authors whom we identified as having no potential conflicts had undeclared conflicts or developed conflicts after the period we examined. One author, Nanette Wenger, for whom we identified no potential conflicts with hormone manufacturers during our study, later declared potential conflicts [37].

Our sample size of ten authors was small, although the authors assessed had written one out of five of the articles identified in our search. The prevalence of financial conflicts among authors in general is unknown, and our findings may not reflect the universe of authors. It is possible that an assessment that included less prolific authors would come to a different conclusion.

Almost all articles were evaluated as scientifically accurate regarding the effect of hormones on breast cancer diagnosis and cardiovascular risk, but readers found phrasing that minimized the risk of breast cancer or seemed to encourage reliance upon animal studies, observational studies, or expert recommendations rather than on randomized controlled trials. Our results support an in-depth interview study that found that physicians at two health plans commonly believed that WHI “was not applicable to the full range of patients seen in clinical practice” and “created uncertainty about the risks and benefits of HT” [38].

Our results suggest that authors who have received payments from industry convey more enthusiasm about the industry's products than do authors who have not declared that they received such payments. These results support the findings of a study that examined conflicts of interest in reviews (among other publications) and found that articles by authors with potential financial conflicts of interest were more likely to support the use of a specific class of drug therapy [39]. The question of whether positive feelings about hormone therapy preceded payments from industry and were perhaps a basis for selection of these physicians as speakers and consultants or whether selection as a speaker or consultant led to more positive feelings about hormone therapy is an issue that should be explored in further research.

Our findings also support an analysis by Tatsioni and colleagues of “partisan editorializing articles on HRT” in the Thomson ISI database by five editorialists who had written at least 12 commentaries in medical journals between 2002 and 2008 [40]. All five had financial relationships with hormone manufacturers; these relationships were reported in only six of the 110 articles analyzed. Although there is no overlap in the author list between the Tatsioni analysis and ours, Tatsioni and colleagues identified similar themes, noting that common arguments included “HRT is effective for menopausal and related symptoms”; “Discussion of preclinical data that showed favorable effects for HRT”; “Statements challenging/criticizing unfavorable studies” (especially against the WHI and the Million Women Study); and “Statements that HRT may decrease life-threatening and other serious outcomes.” Additionally, Tatsioni et al. note that text was sometimes repeated verbatim in several articles; examples are provided in their online supplemental materials [40].

A scientist who consults for the pharmaceutical industry has described the process by which companies formulate key marketing messages into a product narrative to affect the discourse of medicine and ultimately medical knowledge [41]. Although promotional linguistic and rhetorical strategies have been identified in television commercials for prescription drugs [42], there is a dearth of academic articles on the use of rhetoric and persuasion in medical journal articles. To our knowledge, the study by Tatsioni et al. and our study are the first to attempt to assess tone in review articles published in medical journals.

The extent of text reuse we identified was surprising. Tatsioni et al. documented different examples of text repeated verbatim in articles on menopausal hormone therapy, raising the question of how many more articles in the medical literature contain previously published passages. An editorial in *The Lancet* noted that text recycling in review material could be viewed as “less of a crime” than “self-plagiarism” of original research, but that the practice “constitutes intellectual laziness at best” and is unacceptable [43].

### Limitations

The methodology used to evaluate promotional tone for this study has not been previously validated. Our evaluators were not physicians and it is possible that the use of physician evaluators would have yielded different results.

It is possible that the authors for whom no conflicts of interest were found actually did have conflicts of interest, either because we failed to identify a conflict or because a conflict was not disclosed. Misclassification of conflicted authors would be expected to bias the study results toward the null and is unlikely to be responsible for the difference in tone that we identified.

We cannot be certain that the ten authors we evaluated were representative of the universe of authors writing review articles on hormone therapy during the study period. We selected these authors because they were responsible for 20% of the relevant literature during the time period, but a study of authors with fewer publications during the period may have revealed different results. We also did not assess possible conflicts of interest of coauthors or the contribution these coauthors may have made to the accuracy or tone of the articles we assessed.

The assessment of multiple articles by each author may introduce an overcounting problem in the statistical analysis inasmuch as each author's perspective might be expected to stay the same. However, as can be seen in Table 1, about a quarter of articles by authors with potential financial conflicts of interest were deemed nonpromotional, and about a quarter of articles by those without potential financial conflicts of interest were deemed promotional.

Documents recently disclosed in litigation against manufacturers of hormone therapy revealed that dozens of articles ghostwritten by industry were published in the medical literature [44],[45]. The names of two of the authors whose work we assessed, Rogerio Lobo and Leon Speroff, were on the bylines of some of the reportedly ghostwritten articles [45]–[55]. We could not determine whether or not any of the articles assessed in our study were ghostwritten.

### Conclusion

Our study found that narrative review articles on hormone therapy may provide accurate statements about the risks of a therapy while simultaneously providing positive impressions of that therapy for uses unsupported by evidence. There may be a connection between industry funding for research, speaking, or consulting and the publication of promotional pieces on menopausal hormone therapy. Health care providers should exercise caution if they choose to read such articles. We believe that medical journals should follow the International Committee of Medical Journal Editors Uniform Requirements for Manuscripts (http://www.icmje.org/urm_main.html), which require that all authors submit signed statements of their participation in authorship and full disclosure of any conflicts of interest. In order to prevent the bloating of journals with pages of “recycled” text, medical journals should consider using antiplagiarism software.

## Supporting Information

Text S1 Articles assessed.
(0.09 MB PDF)

## Abbreviations

CI: confidence interval
WHI: Women's Health Initiative

## References

[1] Power ML, Schulkin J, Rossouw JE (2007). Evolving practice patterns and attitudes toward hormone therapy of obstetrician-gynecologists.. Menopause.

[2] Power ML, Baron J, Schulkin J (2008). Factors associated with obstetrician-gynecologists' response to the Women's Health Initiative trial of combined hormone therapy.. Med Decis Making.

[3] Power ML, Anderson BL, Schulkin J (2009). Attitudes of obstetrician-gynecologists toward the evidence from the Women's Health Initiative hormone therapy trials remain generally skeptical.. Menopause.

[4] Rossouw JE, Anderson GL, Prentice RL, LaCroix AZ, Kooperberg C (2002). Risks and benefits of estrogen plus progestin in healthy postmenopausal women: Principal results from the Women's Health Initiative randomized controlled trial.. JAMA.

[5] The Women's Health Initiative Steering C (2004). Effects of conjugated equine estrogen in postmenopausal women with hysterectomy: The Women's Health Initiative randomized controlled trial.. JAMA.

[6] Shumaker SA, Legault C, Kuller L, Rapp SR, Thal L (2004). Conjugated equine estrogens and incidence of probable dementia and mild cognitive impairment in postmenopausal women: Women's Health Initiative Memory Study.. JAMA.

[7] Hendrix SL, Cochrane BB, Nygaard IE, Handa VL, Barnabei VM (2005). Effects of estrogen with and without progestin on urinary incontinence.. JAMA.

[8] Hays J, Ockene JK, Brunner RL, Kotchen JM, Manson JE (2003). Effects of estrogen plus progestin on health-related quality of life.. N Engl J Med.

[9] Brunner RL, Gass M, Aragaki A, Hays J, Granek I (2005). Effects of conjugated equine estrogen on health-related quality of life in postmenopausal women with hysterectomy: Results from the Women's Health Initiative randomized clinical trial.. Arch Intern Med.

[10] Majumdar SR, Almasi EA, Stafford RS (2004). Promotion and prescribing of hormone therapy after report of harm by the Women's Health Initiative.. JAMA.

[11] Ravdin PM, Cronin KA, Howlader N, Berg CD, Chlebowski RT (2007). The decrease in breast-cancer incidence in 2003 in the United States.. N Engl J Med.

[12] Chlebowski RT, Kuller LH, Prentice RL, Stefanick ML, Manson JE (2009). Breast cancer after use of estrogen plus progestin in postmenopausal women.. N Engl J Med.

[13] Chlebowski RT, Anderson GL, Gass M, Lane DS, Aragaki AK (2010). Estrogen plus progestin and breast cancer incidence and mortality in postmenopausal women.. JAMA.

[14] Stafford RS, Saglam D, Causino N, Blumenthal D (1997). Low rates of hormone replacement in visits to United States primary care physicians.. Am J Obstet Gynecol.

[15] Saver BG, Taylor TR, Woods NF, Stevens NG (1997). Physician policies on the use of preventive hormone therapy.. Am J Prev Med.

[16] Brett AS, Carney PI, McKeown RE (2005). Brief report: Attitudes toward hormone therapy after the Women's Health Initiative: a comparison of internists and gynecologists.. J Gen Intern Med.

[17] Spangler L, Reed SD, Nekhyludov L, Grothaus LC, LaCroix AZ (2009). Provider attributes associated with hormone therapy prescribing frequency.. Menopause.

[18] Hersh AL, Stefanick ML, Stafford RS (2004). National use of postmenopausal hormone therapy: Annual trends and response to recent evidence.. JAMA.

[19] Fugh-Berman A, Scialli AR (2006). Gynecologists and estrogen: An affair of the heart.. Perspect Biol Med.

[20] Singer N (2009). Medical papers by ghostwriters pushed therapy.. The New York Times.

[21] Ross JS, Hill KP, Egilman DS, Krumholz HM (2008). Guest authorship and ghostwriting in publications related to rofecoxib.. JAMA.

[22] Steinman MA, Bero LA, Chren M, Landefeld CS (2006). Narrative review: the promotion of gabapentin: an analysis of internal industry documents.. Ann Intern Med.

[23] Healy D, Cattel D (2003). Interface between authorship, industry, and science in the domain of therapeutics.. Brit J Psych.

[24] Fugh-Berman A, Melnick D (2008). Off-label promotion, on-target sales.. PLoS Med.

[25] Fauber J, Rust S (2009). UW course for doctors pushed risky therapy.. Milwaukee Journal Sentinel.

[26] Fauber J, Kissinger M (2009). UW linked to ghostwriting.. Milwaukee Journal Sentinel.

[27] Publication Program Drug Industry Document Archive.. http://dida.library.ucsf.edu/tid/kjb37b10.

[28] Publication Program A Proposal for Wyeth-Ayerst Pharmaceuticals - Council on Hormone Education, Scientific Update on Hormones and Postmenopausal Health.. http://dida.library.ucsf.edu/tid/iyb37b10.

[29] Randolph JJ (2008). Online kappa calculator.. http://justus.randolph.name/kappa.

[30] Lexchin J, Bero LA, Djulbegovic B, Clark O (2003). Pharmaceutical industry sponsorship and research outcome and quality: Systematic review.. BMJ.

[31] Jørgensen AW, Hilden J, Gøtzsche PC (2006). Cochrane reviews compared with industry supported meta-analyses and other meta-analyses of the same drugs: Systematic review.. BMJ.

[32] Jørgensen AW, Maric KL, Tendal B, Faurschou A, Gøtzsche PC (2008). Industry-supported meta-analyses compared with meta-analyses with non-profit or no support: differences in methodological quality and conclusions.. BMC Med Res Methodol.

[33] Cosgrove L, Bursztajn HJ, Krimsky S, Anaya M, Walker J (2009). Conflicts of interest and disclosure in the American Psychiatric Association's Clinical Practice Guidelines.. Psychother Psychosom.

[34] Rose J (2008). Industry influence in the creation of pay-for-performance quality measures.. Qual Manag Health Care.

[35] Hemminki E (2003). Opposition to unpopular research results: Finnish professional reactions to the WHI findings.. Health Policy.

[36] Prentice RL, Chlebowski RT, Stefanick ML, Manson JE, Pettinger M (2008). Estrogen plus progestin therapy and breast cancer in recently postmenopausal women.. Am J Epidemiol.

[37] American College of Cardiology ACCEL: ACCF's Disclosure/conflict of interest policy.. http://stage.acc.org/accel_disclosures.htm.

[38] Bush TM, Bonomi AE, Nekhlyudov L, Ludman EJ, Reed SD (2007). How the Women's Health Initiative (WHI) influenced physicians' practice and attitudes.. J Gen Intern Med.

[39] Stelfox HT, Chua G, O'Rourke K, Detsky AS (1998). Conflict of interest in the debate over calcium-channel antagonists.. N Engl J Med.

[40] Tatsioni A, Siontis GCM, Ioannidis JPA (2010). Partisan perspectives in the medical literature: A study of high frequency editorialists favoring hormone replacement therapy.. J Gen Int Med.

[41] Matheson A (2008). Corporate science and the husbandry of scientific and medical knowledge by the pharmaceutical industry.. BioSocieties.

[42] Glinert LH (2005). TV commercials for prescription drugs: a discourse analytic perspective.. Res Social Admin Pharm.

[43] [No authors listed] (2009). Self-plagiarism: Unintentional, harmless, or fraud?. Lancet.

[44] PLoS Medicine (2009). Ghostwriting: The dirty little secret of medical publishing that just got bigger.. http://www.plosmedicine.org/article/info%3Adoi%2F10.1371%2Fjournal.pmed.1000156.

[45] Fugh-Berman AJ (2010). The haunting of medical journals: How ghostwriting sold “HRT”.. PLoS Med.

[46] Draft of Low-Dose Review Paper [With Annotations] DWRITE073011. Prempro Products Liability Litigation, MDL Docket No 4:03CV1507 WRW (W.D. Arkansas).. http://dida.library.ucsf.edu/tid/qlc37b10.

[47] Low-dose Review Paper Submitted to Climacteric DWRITE73106. Prempro Products Liability Litigation, MDL Docket No 4:03CV1507 WRW (W.D. Arkansas).. http://dida.library.ucsf.edu/tid/qnb37b10.

[48] Response Letter to Alastair MacLennan Prempro Products Liability Litigation, MDL Docket No 4:03CV1507 WRW (W.D. Arkansas). DWRITE072945.. http://dida.library.ucsf.edu/tid/qib37b10.

[49] Draft of metabolic paperDELCA032-028548 Prempro Products Liability Litigation, MDL Docket No 4:03CV1507 WRW (W.D. Arkansas).. http://dida.library.ucsf.edu/tid/czb37b10.

[50] Effects of Lower Doses of Conjugated Estrogens and Medroxyprogesterone Acetate on Plasma Lipids and Lipoproteins, Coagulation Factors, and Carbohydrate Metabolism Prempro Products Liability Litigation, MDL Docket No 4:03CV1507 WRW (W.D. Arkansas). DELCA032-028549.. http://dida.library.ucsf.edu/tid/czc37b10.

[51] Inconsistency in Epidemiological Findings on Postmenopausal Hormone Therapy and Breast Cancer DWRITE078512. (Exh. 96 to Janas Depos.) Prempro Products Liability Litigation, MDL Docket No 4:03CV1507 WRW (W.D. Arkansas).. http://dida.library.ucsf.edu/tid/rrb37b10.

[52] Re: Draft of Manuscript DWRITE078370. (Exh. 95 to Janas Depos.) Prempro Products Liability Litigation, MDL Docket No 4:03CV1507 WRW (W.D. Arkansas).. http://dida.library.ucsf.edu/tid/rqc37b10.

[53] Janas Deposition: 483:1l–485:13 Prempro Products Liability Litigation, MDL Docket No 4:03CV1507 WRW (W.D. Arkansas).. http://dida.library.ucsf.edu/pdf/skc37b10.

[54] Endometrial paper and action steps from last meeting DELCA031-019050 Prempro Products Liability Litigation, MDL Docket No 4:03CV1507 WRW (W.D. Arkansas).. http://dida.library.ucsf.edu/search?query=DELCA031-019050&ct=1.

[55] Endometrial Effects of Lower Doses of Conjugated Equine Estrogens and Medroxyprogesterone Acetate DELCA031-019052. Prempro Products Liability Litigation, MDL Docket No 4:03CV1507 WRW (W.D. Arkansas).. http://dida.library.ucsf.edu/pdf/cvc37b10.

