# Supplementary material for: Promotional Tone in Reviews of Menopausal Hormone Therapy After the Women's Health Initiative: An Analysis of Published Articles
Source: PLoS Med. 2011 Mar 15;8(3):e1000425. doi: 10.1371/journal.pmed.1000425 (PMC3058057; doi:10.1371/journal.pmed.1000425)
Supplement: Text S1 — Articles assessed. (0.09 MB PDF) [file pmed.1000425.s001.pdf]

Text S1 - Articles Assessed

A1. Chlebowski RT, Col N (2004) Menopausal hormone therapy after breast cancer. Lancet 363: 410-411.

A2. Chlebowski RT, Anderson GL (2005) Progestins and Recurrence in Breast Cancer Survivors. J Natl Cancer Inst 97: 471-472.

A3. Col NF (2006) Decision making at the fringe of evidence: take what you can get. Arch Intern Med 166: 389-390.

A4. Col NF (2005) The impact of risk status, preexisting morbidity, and polypharmacy on treatment decisions concerning menopausal symptoms. Am J Med 118 Suppl 12B: 155-162.

A5. Herrington DM, Howard TD (2003) From presumed benefit to potential harm--hormone therapy and heart disease. N Engl J Med 349: 519-521.

A6. Wenger NK (2004) Diet and exercise for perimenopausal women lifestyle interventions can decrease cardiovascular risk. J Am Coll Cardiol 44: 586-587.

A7. Paoletti R, Wenger NK (2003) Review of the International Position Paper on Women's Health and Menopause: a comprehensive approach. Circulation 107: 1336-1339.

A8. Speroff L (2003) The Million Women Study and breast cancer. Maturitas 46: 1-6.

A9. Speroff L (2004) Postmenopausal hormone therapy and the risk of breast cancer. A clinician's view. Maturitas 49: 51-57.

A10. Speroff L (2005) Clinical appraisal of the Women's Health Initiative. J Obstet Gynaecol Res 31: 80-93.

A11. Kenemans P (2005) Postmenopausal hormone therapy and breast cancer: what is the problem? Maturitas 51: 75-82.

A12. Brood-van Zanten MM, Barentsen R, van der Mooren MJ (2002) Hormone replacement therapy and surveillance considerations. Maturitas 43 Suppl 1: S57-67.

A13. Wenger NK (2005) Menopausal hormone therapy: currently no evidence for cardiac protection. Pediatr Blood Cancer 44: 625-629.

A14. Lobo RA (2005) Appropriate use of hormones should alleviate concerns of cardiovascular and breast cancer risk. Maturitas 51: 98-109.

A15. Herrington DM, Klein KP (2003) Randomized clinical trials of hormone replacement therapy for treatment or prevention of cardiovascular disease: a review of the findings. Atherosclerosis 166: 203-212.

A16. Herrington DM, Parks JS (2004) Estrogen and HDL: all that glitters is not gold. Arterioscler Thromb Vasc Biol 24: 1741-1742.

A17. Kenemans P, Bosman A (2003) Breast cancer and post-menopausal hormone therapy. Best Pract Res Clin Endocrinol Metab 17: 123-137.

A18. van der Mooren MJ, Kenemans P (2003) Postmenopausal hormone replacement therapy in the light of the women's health initiative trial. Eur J Obstet Gynecol Reprod Biol 107: 123-124.

A19. Grimes DA, Lobo RA (2002) Perspectives on the Women's Health Initiative trial of hormone replacement therapy. Obstet Gynecol 100: 1344-1353.

A20. van der Mooren MJ, Kenemans P (2004) Postmenopausal hormone therapy: impact on menopause-related symptoms, chronic disease and quality of life. Drugs 64: 821-836.

A21. Clarkson TB, Appt SE (2005) Controversies about HRT--lessons from monkey models. Maturitas 51: 64-74.

A22. Clarkson TB, Appt SE (2003) MPA and postmenopausal coronary artery atherosclerosis revisited. Steroids 68: 941-951.

A23. Mikkola TS, Clarkson TB (2006) Coronary heart disease and postmenopausal hormone therapy: conundrum explained by timing? J Womens Health 15: 51-53.

A24. Hickey M, Davis SR, Sturdee DW (2005) Treatment of menopausal symptoms: what shall we do now? Lancet 366: 409-421.

A25. Davison S, Davis SR (2003) Hormone replacement therapy: current controversies. Clin Endocrinol 58: 249-261.

A26. Davis SR, Dinatale I, Rivera-Woll L, Davison S (2005) Postmenopausal hormone therapy: from monkey glands to transdermal patches. J Endocrinol 185: 207-222.

A27. Wagner JD, Clarkson TB (2005) The applicability of hormonal effects on atherosclerosis in animals to heart disease in postmenopausal women. Semin Reprod Med 23: 149-156.

A28. Wenger NK (2003) Coronary heart disease: the female heart is vulnerable. Prog Cardiovasc Dis 46: 199-229.

A29. Speroff L (2005) Postmenopausal hormone therapy and cardiovascular disease: one view of the elephant. Menopause 12: 357-358.

A30. Speroff L (2004) Postmenopausal hormone therapy and breast cancer: a clinician's message for patients. Endocrine 24: 211-216.

A31. Mikkola TS, Clarkson TB, Notelovitz M (2004) Postmenopausal hormone therapy before and after the women's health initiative study: what consequences? Ann Med 36: 402-413.

A32. Herrington DM, Howard TD (2003) ER-alpha variants and the cardiovascular effects of hormone replacement therapy. Pharmacogenomics 4: 269-277.

A33. Lobo RA (2004) The rationale for low-dose hormonal therapy. Endocrine 24: 217-221.

A34. Kenemans P (2005) Women's health after WHI. Maturitas 51: 1-3.

A35. Genazzani AR, Gambaciani M, vand der Mooren MJ, Speroff L, Doren M, et al. (2003) Critical comments. Maturitas 44: 11-18.

A36. Speroff L, Kenemans P, Burger HG (2005) Practical guidelines for postmenopausal hormone therapy. Maturitas 51: 4-7.

A37. van der Mooren MJ, Kenemans P (2004) The Million Women Study: a licence to kill other investigations? Eur J Obstet Gynecol Reprod Biol 113: 3-5.

A38. Clarkson TB, Appt SE, Wood CE, Cline JM (2004) Lessons to be learned from animal studies on hormones and the breast. Maturitas 49: 79-89.

A39. Col NF, Pauker SG (2003) The discrepancy between observational studies and randomized trials of menopausal hormone therapy: did expectations shape experience? Ann Intern Med 139: 923-929.

A40. Burger H, Archer D, Barlow D, Birkhauser M, Calaf-Alsina J, et al. (2004) Practical recommendations for hormone replacement therapy in the peri- and postmenopause. Climacteric 7: 210-216.

A41. Lobo RA (2004) Evaluation of cardiovascular event rates with hormone therapy in healthy, early postmenopausal women: results from 2 large clinical trials. Arch Intern Med 164: 482-484.

A42. Miller VM, Clarkson TB, Harman SM, Brinton EA, Cedars M, et al. (2005) Women, hormones, and clinical trials: a beginning, not an end. J Appl Physiol 99: 381-383.

A43. Naftolin F, Taylor HS, Karas R, Brinton E, Newman I, et al. (2004) The Women's Health Initiative could not have detected cardioprotective effects of starting hormone therapy during the menopausal transition. Fertil Steril 81: 1498-1501.

A44. Wenger NK (2004) Menopausal hormone therapy: is there evidence for cardiac protection? Int Urol Nephrol 36: 617-623.

A45. Speroff L (2003) WHI trial: it's time to be critical! Am J Obstet Gynecol 189: 620.

A46. Klein KP, Herrington DM (2002) Effects of estrogens and selective estrogen receptor modulators on indicators of cardiovascular health in postmenopausal women. Medscape Womens Health 7: 2.

A47. Chlebowski RT, Kim JA, Col NF (2003) Estrogen deficiency symptom management in breast cancer survivors in the changing context of menopausal hormone therapy. Semin Oncol 30: 776-788.

A48. Vogelvang TE, van der Mooren MJ, Mijatovic V (2004) Hormone replacement therapy, selective estrogen receptor modulators, and tissue-specific compounds: cardiovascular effects and clinical implications. Treat Endocrinol 3: 105-115.

A49. Wenger NK (2003) Menopausal hormone therapy and cardiovascular protection: state of the data 2003. J Am Med Womens Assoc 58: 236-239.

A50. Speroff L (2004) A clinician's review of the WHI-related literature. Int J Fertil Womens Med 49: 252-267.
